# Taxifolin Prevents Cisplatin Nephrotoxicity by Modulating Nrf2/HO-1 Pathway and Mitigating Oxidative Stress and Inflammation in Mice

**DOI:** 10.3390/ph15111310

**Published:** 2022-10-24

**Authors:** Abdulkareem A. Alanezi, Afaf F. Almuqati, Manal A. Alfwuaires, Fawaz Alasmari, Nader I. Namazi, Osama Y. Althunibat, Ayman M. Mahmoud

**Affiliations:** 1 Department of Pharmaceutics, College of Pharmacy, University of Hafr Al-Batin, Hafr Al-Batin 31991, Saudi Arabia; 2 Department of Pharmaceutical Chemistry, College of Pharmacy, University of Hafr Al-Batin, Hafr Al-Batin 31991, Saudi Arabia; 3 Department of Biological Sciences, Faculty of Science, King Faisal University, Al-Ahsa 31982, Saudi Arabia; 4 Department of Pharmacology and Toxicology, College of Pharmacy, King Saud University, Riyadh 11451, Saudi Arabia; 5 Pharmaceutics and Pharmaceutical Technology Department, College of Pharmacy, Taibah University, Al Madinah Al Munawarah 30001, Saudi Arabia; 6 Department of Medical Analysis, Princess Aisha Bint Al-Hussein College of Nursing and Health Sciences, Al-Hussein Bin Talal University, Ma’an 71111, Jordan; 7 Physiology Division, Zoology Department, Faculty of Science, Beni-Suef University, Beni-Suef 62514, Egypt; 8 Department of Life Sciences, Faculty of Science and Engineering, Manchester Metropolitan University, Manchester M1 5GD, UK

**Keywords:** kidney injury, chemotherapy, flavonoids, Nrf2, oxidative stress

## Abstract

Cisplatin (CIS) is an effective chemotherapeutic agent used in the treatment of several malignancies. The clinical use of CIS is associated with adverse effects, including acute kidney injury (AKI). Oxidative stress and inflammation are key events in the development of CIS-induced AKI. This study investigated the protective effect of taxifolin (TAX), a bioactive flavonoid with promising health-promoting properties, on CIS-induced nephrotoxicity in mice. TAX was orally given to mice for 10 days and a single dose of CIS was injected at day 7. Serum blood urea nitrogen (BUN) and creatinine were elevated, and multiple histopathological alterations were observed in the kidney of CIS-administered mice. CIS increased renal malondialdehyde (MDA), nitric oxide (NO), nuclear factor-kappaB (NF-κB) p65, tumor necrosis factor (TNF)-α, and interleukin (IL)-1β, and decreased cellular antioxidants in mice. TAX remarkably prevented kidney injury, ameliorated serum BUN and creatinine, and renal MDA, NO, NF-κB p65, and pro-inflammatory cytokines, and boosted antioxidant defenses in CIS-administered mice. TAX downregulated Bax and caspase-3, and upregulated Bcl-2. These effects were associated with upregulation of nuclear factor erythroid 2-related factor 2 (Nrf2) expression and heme oxygenase (HO)-1 activity in CIS-administered mice. In conclusion, TAX prevented CIS-induced AKI by mitigating tissue injury, oxidative stress, inflammation, and cell death. The protective efficacy of TAX was associated with the upregulation of Nrf2/HO-1 signaling.

## 1. Introduction

Cisplatin (CIS) is one of the most potent chemotherapy agents widely used for the treatment of many malignancies, including testicular, ovarian, and bladder cancers [1,2]. It is a platinum-based cytotoxic agent that increases the generation of reactive oxygen species (ROS) and interferes with DNA synthesis through crosslinking, leading to cell death [3]. The use of CIS is associated with several adverse effects, including acute kidney injury (AKI) which can be life-threatening [4,5], thereby compromising its clinical applications. The kidneys are vulnerable to toxicity and AKI occurs in 20–35% of patients after treatment with CIS which is absorbed into renal tubular cells where it can accumulate and cause tubular injury [6]. The mechanism underlying CIS nephrotoxicity is complex and not fully understood; however, oxidative stress and inflammation have been significantly implicated [6]. CIS provokes ROS generation depending on both the concentration and the exposure time [7]. Excess ROS can inflict cellular damage, including lipid peroxidation (LPO) and oxidative damage of DNA and protein. In addition, ROS activates nuclear factor-kappaB (NF-κB) which can promote the expression of a variety of pro-inflammatory mediators. Excess ROS and inflammatory mediators work in concert to induce cell death via apoptosis, and inflammation is strongly correlated with renal failure induced by CIS [8]. Therefore, attenuation of ROS and inflammation could be an effective strategy to protect against CIS-induced AKI.

Activation of the nuclear factor erythroid 2-related factor 2 (Nrf2) promotes the expression of genes that are effective in mitigating ROS and inflammation [9]. Several reports have demonstrated the beneficial effects conferred by Nrf2 activation against drug-induced kidney injury [10,11,12,13], whereas its deficiency augmented AKI induced by ischemia or nephrotoxic drugs [14]. Nrf2 is a transcription factor that is found sequestered by Kelch-like ECH-associated protein 1 (Keap1) in the cytosol under physiological conditions, a binding that dissociates upon exposure to ROS or electrophiles. The liberated Nrf2 translocate into the nucleus, where it binds the antioxidant response element (ARE) and elicits the expression of heme oxygenase (HO)-1, superoxide dismutase (SOD), and other antioxidant genes [9].

Flavonoids are polyphenolic plant secondary metabolites with antioxidant and anti-inflammatory activities [15,16,17,18]. Accumulating evidence indicates that numerous flavonoids have demonstrated therapeutic and preventive properties against CIS nephrotoxicity through modulating ROS, inflammatory responses, and Nrf2/HO-1 signaling [19,20,21]. Taxifolin (TAX; 3,5,7,3,4-pentahydroxy flavanone) is a flavonoid found in many plants and showed various pharmacological prosperities such as antioxidant, anti-cancer, and anti-inflammatory [22,23,24]. TAX alleviated CIS-induced pulmonary injury in rats through attenuation of oxidative stress and restoration of antioxidants [25], and ameliorated renal injury in a murine model of unilateral ureteral obstruction (UUO) by mitigating oxidative stress, inflammatory responses, and fibrosis in rats [26]. In human retinal pigment epithelium (RPE), TAX protected against oxidative stress-induced cellular damage and apoptosis via activation of Nrf2 and the phase II antioxidant enzyme system [24]. Moreover, TAX ameliorated acute alcohol-induced hepatic injury by suppressing oxidative stress and NF-κB-mediated inflammatory reaction [27]. The activation of peroxisome proliferator-activated receptor γ coactivator-1α [28] and enhanced antioxidants [28,29] contributed to the protective effect of TAX against chemotherapy-induced nephrotoxicity in rodents. Despite its various pharmacological properties, the protective effect of TAX against CIS nephrotoxicity is not fully understood. This study aimed to investigate the effect of TAX on CIS-induced AKI, pointing to its ability to modulate Nrf2/HO-1 signaling and attenuate oxidative stress, inflammation, and apoptosis.

## 2. Results

### 2.1. TAX Prevents CIS-Induced Body Weight Loss and Kidney Injury in Mice

**Figure 1:**
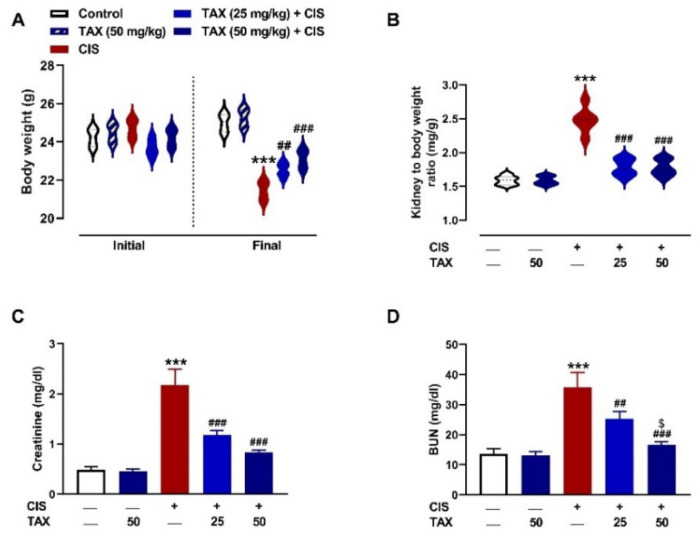
TAX prevented body weight loss (**A**), and decreased kidney weight–to–body weight ratio (**B**), serum creatinine (**C**), and BUN (**D**) in CIS-intoxicate mice. Data are mean ± SEM, (*n* = 6). *** *p* < 0.001 versus Control, ^##^ *p* < 0.01 and ^###^ *p* < 0.001 versus CIS, and ^$^ *p* < 0.05 versus TAX (25 mg/kg).

**Figure 2:**
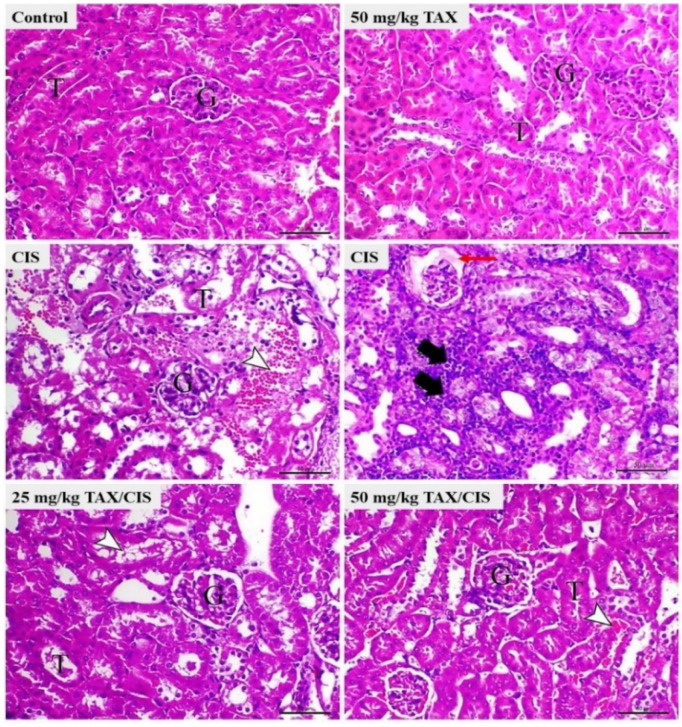
Photomicrographs of sections in the kidney of control and TAX-treated mice showing normal renal glomeruli (G) and tubules (T), CIS-intoxicated mice showing parenchymal haemorrhage (arrowhead) and a severe degree of vacuolar degnerative changes within the lining epithelium of the renal tubules, enlarged space around glomeruli (red arrow), and inflammatory cell infiltration (black arrow), CIS-administered mice treated with 25 mg/kg TAX showing a decrease in the vacuolar degenerative changes (arrowhead), and 50 mg/kg TAX, showing mild congestion of the renal blood cappillaries (arrowhead) and marked decrease in the degenerative changes within the lining epithelium of the renal tubules. (Hematoxylin and eosin (H & E), scale bar = 50 µm).

**Figure 3:**
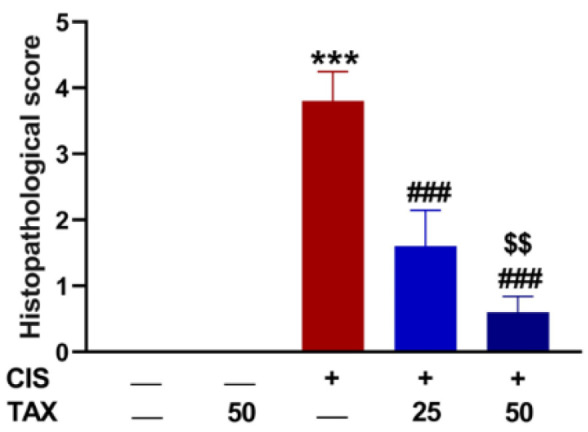
Histopathological scores of kidney sections of mice. TAX decreased kidney injury score in CIS-intoxicated mice. Data are mean ± SEM. *** *p* < 0.001 versus Control, ^###^ *p* < 0.001 versus CIS, and ^$$^ *p* < 0.001 versus TAX (25 mg/kg).

To investigate the protective effect of TAX on CIS-induced renal injury, we assessed the kidney weight–to–body weight ratio, kidney function biomarkers (Figure 1), and histopathological changes (Figure 2 and Figure 3). Mice that received CIS exhibited a significant (*p* < 0.001) decrease in body weight (Figure 1A) and increase in kidney weight–to–body weight ratio (Figure 1B), serum creatinine (Figure 1C) and blood urea nitrogen (BUN) (Figure 1D). Both doses of TAX prevented body weight loss and ameliorated serum BUN and creatinine in CIS-administered mice with the higher dose being more effective in decreasing urea. TAX alone had no effect on these markers in normal mice. These biochemical findings were supported by the microscopic examination as shown in Figure 2 and Figure 3. Examination of kidney sections of control and TAX-treated mice revealed normal renal tissue appearance with typical glomeruli and tubule structure. Sections in the kidney of CIS-intoxicated mice revealed vacuolar degenerative changes of the tubular epithelium, inflammatory cells infiltration, glomerular deformity and atrophy, and parenchymal haemorrhage. Both doses of TAX prevented these CIS-induced alterations in the kidney of mice.

### 2.2. TAX Attenuates CIS-Induced Renal Oxidative Stress in Mice

**Figure 4:**
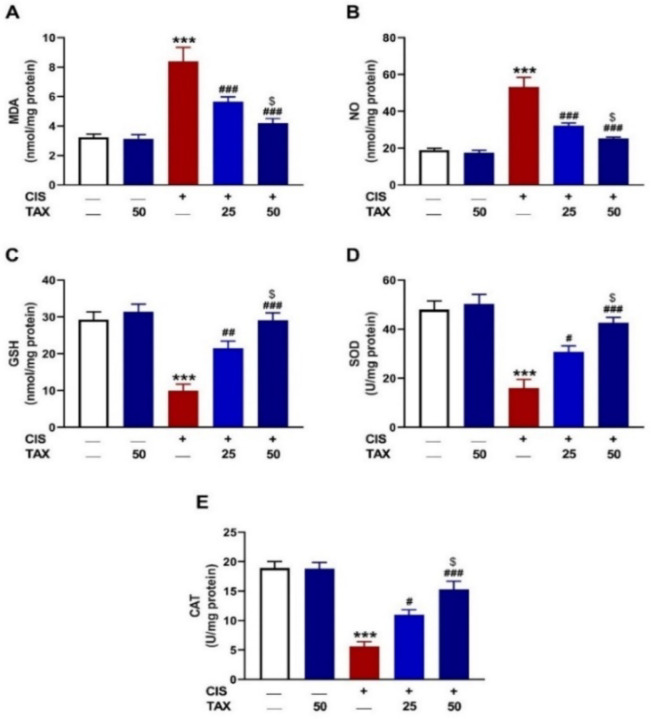
TAX attenuated CIS-induced renal oxidative stress. TAX decreased (**A**) MDA and (**B**) NO, and increased (**C**) GSH, (**D**) SOD, and (**E**) CAT in the kidney of CIS-administered mice. Data are mean ± SEM, (*n* = 6). *** *p* < 0.001 versus Control, ^#^ *p* < 0.05, ^##^ *p* < 0.01 and ^###^ *p* < 0.001 versus CIS, and ^$^ *p* < 0.05 versus TAX (25 mg/kg).

Malondialdehyde (MDA), nitric oxide (NO), and antioxidants were determined to evaluate the efficacy of TAX against CIS-induced redox imbalance. CIS-treated mice showed a significant increase in renal MDA (Figure 4A) and NO (Figure 4B), with concomitant decline in reduced glutathione (GSH) (Figure 4C), SOD (Figure 4D), and CAT (Figure 4E) as compared to the control mice (*p* < 0.001). TAX dose-dependently decreased MDA and NO, and enhanced GSH and antioxidant enzymes in CIS-intoxicated mice. TAX had no effect on the assayed parameters when supplemented to normal mice.

### 2.3. TAX mitigates Kidney Inflammation in CIS-Intoxicated Mice

**Figure 5:**
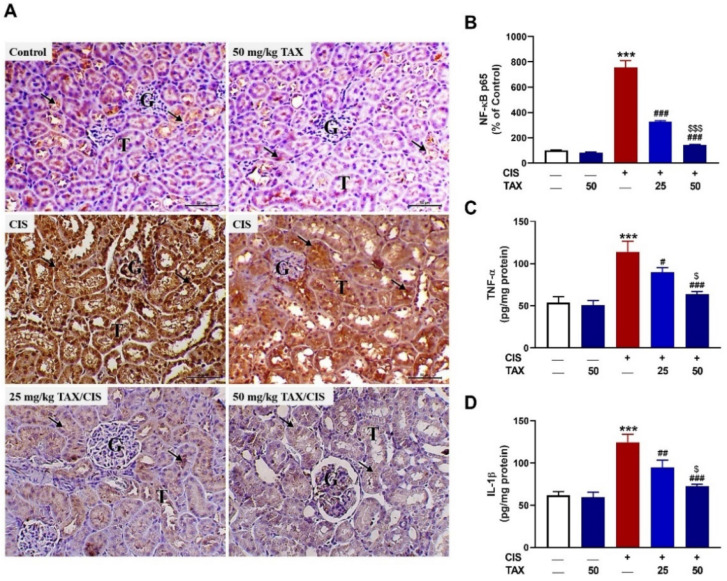
TAX mitigated CIS-induced renal inflammation. (**A**) Photomicrographs of sections in the kidney of control mice showing slight expression of NF-κB p65 within the renal tubular epithelium (arrows), TAX-treated mice showing minimal expression of NF-κB p65, CIS-intoxicated mice showing marked expression of NF-κB p65 (arrows), and CIS-administered mice treated with 25 and 50 mg/kg TAX showing marked decrease in NF-κB p65 (arrows). (G, glomeruli; T, tubules—scale bar = 50 µm). (**B**) Image analysis of NF-κB p65 showing a significant decrease in NF-κB p65 in CIS-administered mice treated with TAX. (C-D) TAX decreased TNF-α (**C**) and IL-1β (**D**) in CIS-administered mice. Data are mean ± SEM, (*n* = 6). *** *p* < 0.001 versus Control, ^#^ *p* < 005, ^##^ *p* < 0.01 and ^###^ *p* < 0.001 versus CIS, and ^$^ *p* < 0.05 and ^$$$^ *p* < 0.001 versus TAX (25 mg/kg).

The inflammatory response plays a key role in the development and progression of CIS-induced renal dysfunction and injury [8]. As shown in Figure 5A,B, there was a significant (*p* < 0.001) increase in NF-κB p65 in the kidney of CIS-administered mice as compared to the control group. Similarly, significant upregulation of TNF-α (Figure 5C) and IL-1β (Figure 5D) in CIS-administered mice was observed (*p* < 0.001). TAX downregulated NF-κB p65, TNF-α, and IL-1β levels in CIS-administered mice in a dose-dependent manner, whereas it had no effect when supplemented to normal mice.

### 2.4. TAX Prevents CIS-Induced Renal Apoptosis

**Figure 6:**
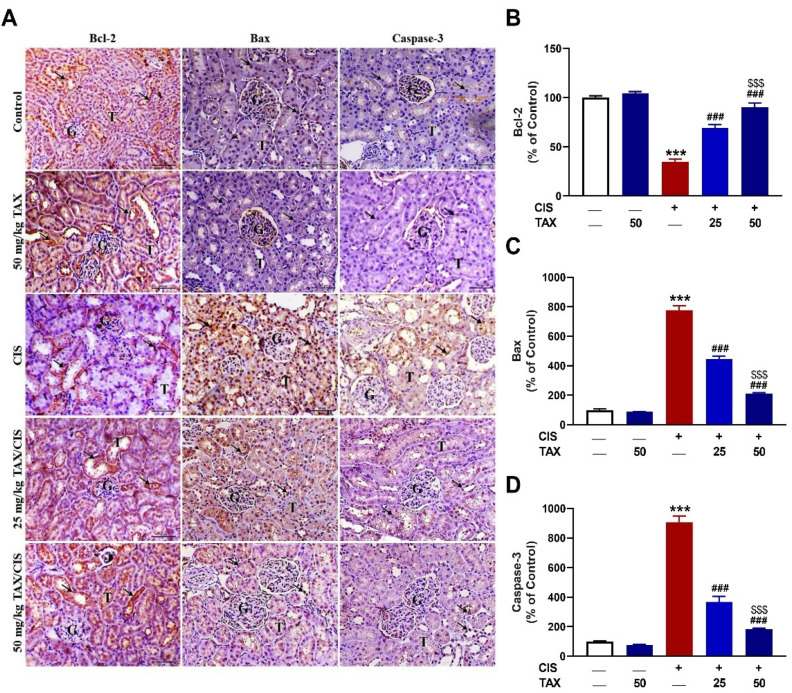
TAX prevented CIS-induced apoptosis in the kidney of mice. (**A**) Photomicrographs of sections in the kidney of control and TAX-treated mice showing marked expression of Bcl-2, and mild expression of Bax and caspase-3 (arrow), of CIS-administered mice showing decreased Bcl-2, and markedly increased Bax and caspase-3 (arrow), and of CIS-administered mice treated with 25 and 50 mg/kg TAX showing marked increase in Bcl-2, and noticeable decrease in Bax and caspase-3 (arrow) (G, glomeruli; T, tubules—scale bar = 50 µm). (**B**–**D**) Image analysis showing significant increase in Bcl-2 and decreased Bax and caspase-3 in CIS-administered mice treated with TAX. Data are mean ± SEM, (*n* = 6). *** *p* < 0.001 versus Control, ^###^ *p* < 0.001 versus CIS, and ^$$$^ *p* < 0.001 versus TAX (25 mg/kg).

To assess the protective impact of TAX on CIS-induced renal apoptosis, we determined the expression levels of Bax, Bcl-2, and caspase-3. There was a significant (*p* < 0.001) decrease in Bcl-2 (Figure 6A,B), an increase in Bax (Figure 6A,C), and cleaved caspase-3 (Figure 6A,D) in the kidney of CIS-administered mice as compared to the control group. TAX dose-dependently increased Bcl-2 and decreased Bax and caspase-3 in the kidney of CIS-administered mice. TAX at a dose level of 50 mg/kg had no effect on the assayed apoptosis markers when supplemented to normal mice.

### 2.5. TAX Upregulates Nrf2/HO-1 Pathway in the Kidney of CIS-Intoxicated Mice

**Figure 7:**
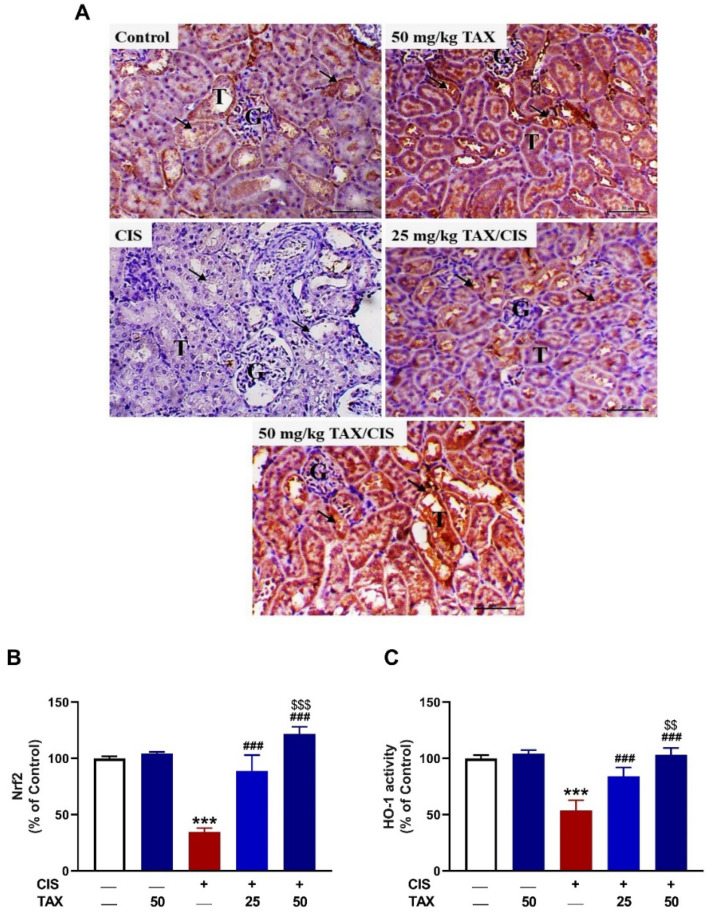
TAX upregulated Nrf2/HO-1 signaling in the kidney of CIS-administered mice. (**A**) Photomicrographs of kidney sections stained with anti-Nrf2 antibody showing marked expression in control and TAX-treated mice, a noticeable decrease in CIS-administered mice, and increased expression in CIS-administered mice treated with 25 and 50 mg/kg TAX. (**B**) Image analysis showing significant increase in Nrf2 in CIS-administered mice treated with TAX. (**C**) TAX enhanced HO-1 activity in the kidney of CIS-administered mice. Data are mean ± SEM, (*n* = 6). *** *p* < 0.001 versus Control, ^###^ *p* < 0.001 versus CIS, and ^$$^ *p* < 0.01 and ^$$$^ *p* < 0.001 versus TAX (25 mg/kg).

CIS provoked a significant downregulation of Nrf2 (Figure 7A,B) and HO-1 activity (Figure 7C) as compared to the control mice (*p* < 0.001). TAX upregulated Nrf2 and HO-1 in the kidney of CIS-administered mice in a dose-dependent manner.

### 2.6. TAX Does Not Interfere with the Anti-Proliferative Activity of CIS

**Figure 8:**
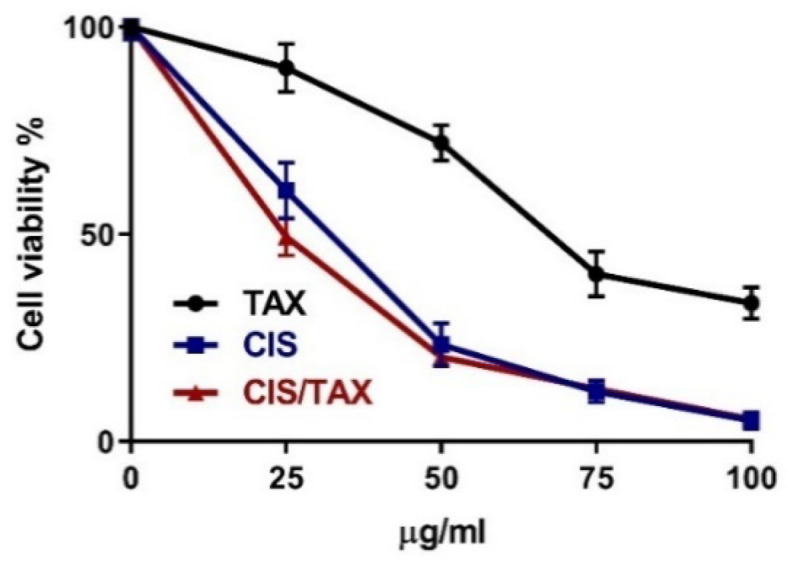
TAX does not interfere with the anti-proliferative activity of CIS. Data are mean ± SEM. The experiment was repeated three times (*n* = 3).

We evaluated the cytotoxic effect of CIS alone and in combination with TAX in HepG2 cells to assess any interference. Treatment of HepG2 cells with either TAX or CIS resulted in cytotoxicity. TAX did not interfere with the cytotoxicity of CIS and the combined treatment resulted in a more potent anti-proliferative activity (Figure 8).

## 3. Discussion

Nephrotoxicity caused by the platinum compound CIS is one of the factors that limit its clinical use in spite of its highly effective therapeutic efficacy against several cancers [4,5,6]. CIS nephrotoxicity involves increased ROS generation and activation of pro-inflammatory and cell death pathways, eventually culminating in kidney injury [6,7]. The use of ROS scavenging compounds has been widely accepted as a promising feasible strategy for the prevention/treatment of chemotherapy-induced toxicity [19,20,21]. TAX is one of the flavonoids that showed effective antioxidant and anti-inflammatory activities [22,23,24] and hence could be effective against CIS-induced AKI. Herein, we investigated the protective efficacy of TAX against CIS-induced oxidative injury and inflammatory response in the kidney of mice.

CIS administration caused nephrotoxicity represented by the elevated serum BUN and creatinine levels as previously reported [30,31]. The biochemical findings were supported by the histopathological alterations, including vacuolar degenerative changes of the tubular epithelium, inflammatory cell infiltration, glomerular deformity and atrophy, and parenchymal haemorrhage. Oral supplementation of TAX prevented CIS-induced tissue injury and ameliorated the circulating kidney function markers, pinpointing its nephroprotective efficacy. Previous studies have demonstrated the ability of TAX to protect the kidney against injury in a rat model of UUO [26] and against nephrotoxicity induced by acrylamide [32] and rotenone [33]. The current findings support the nephroprotective effect of TAX and introduced information that it can attenuate CIS nephrotoxicity.

Given the key role of oxidative injury and inflammation in mediating CIS toxicity and AKI [7,8], we assumed that TAX prevented CIS nephrotoxicity by its dual antioxidant and anti-inflammatory activity. In this study, CIS-intoxicated mice exhibited an increase in renal LPO and NO accompanied with declined GSH and antioxidant enzymes, denoting oxidative stress. CIS-induced ROS production, which could be a result of neutrophil activation and mitochondrial dysfunction [7], which can damage cellular macromolecules, including membrane lipids via peroxidation. LPO alters membrane permeability and fluidity and inactivates membrane-bound proteins, resulting in cell death [34]. NO reacts with superoxide radicals to produce the potent oxidant peroxynitrite that can augment ROS generation, modify DNA bases, and induce DNA breaks and cell death [35]. ROS can also activate the redox-sensitive factor NF-κB that elicits pro-inflammatory responses through the release of pro-inflammatory mediators [36]. Here, CIS promoted renal inflammation manifested by the upregulation of NF-κB p65, TNF-α, and IL-1β as reported in previous studies [19,20,37]. In human [38] and murine [39] AKI, activation of NF-κB was observed and the released cytokines increase ROS in the kidney by activating macrophages and neutrophils [40]. In CIS-intoxicated animals, an increase in renal neutrophil infiltration and ROS has been reported [41,42] and the histopathological findings of the current study revealed inflammatory cell infiltration. Moreover, ROS and inflammatory mediators work in concert to provoke cell death via apoptosis by activating Bax [43]. Our findings revealed an increase in Bax and caspase-3 and decreased Bcl-2 in the kidney of CIS-intoxicated mice. Bax deteriorates mitochondrial membrane potential (MMP) and mitochondrial permeability transition, leading to the release of cytochrome *c* into the cytoplasm. Cytochrome *c* binds procaspase-9, apoptotic peptidase activating factor 1, and ATP to produce the apoptosome complex, which activates caspase-3. Caspase-3 is an executioner enzyme that provokes the degradation of cytoskeletal proteins, DNA fragmentation, and cell death [44]. Bcl-2 is an anti-apoptotic protein that prevents the release of cytochrome *c* into the cytoplasm and the subsequent activation of caspases. Thus, prevention of the CIS-induced ROS overproduction and NF-κB activation can attenuate apoptosis in the kidney.

TAX enhanced antioxidant defenses, suppressed LPO and NF-κB, and decreased NO, TNF-α, and IL-1β in the kidney of CIS-intoxicated mice, demonstrating its antioxidant and anti-inflammatory activities. In addition, TAX mitigated CIS-induced apoptosis in mouse kidney as shown by the decreased Bax and caspase-3 and upregulation of Bcl-2. The antioxidant and anti-inflammatory efficacies of TAX were demonstrated in experimental renal injury induced by UOO in rats, where it mitigated oxidative stress and inflammatory responses [26]. In acrylamide- and rotenone-administered rats, TAX protected the kidney against oxidative injury and inflammation, where it suppressed LPO, TNF-α, and IL-1β, and enhanced GSH [32,33]. TAX suppressed the infiltration of polymorphonuclear leukocytes in the lung of CIS-administered rats, an effect that was accompanied with decreased MDA and enhanced GSH [25]. A very recent study showed ameliorated oxidative stress and inflammation in the kidney of rats challenged with cadmium following treatment with TAX [45]. The anti-apoptosis effect of TAX reported in this study could be directly explained by the attenuation of oxidative stress and inflammation. In support of these findings, a recent study reported that TAX prevented gentamicin-induced apoptosis in mouse cochlear cells by preventing ROS-induced alteration in MMP and ameliorating Bax and Bcl-2 expression [46]. The ability of TAX to prevent oxidative stress and apoptosis has also been reported in human RPE [24] and alcohol-induced acute hepatic injury in mice [27]. Furthermore, TAX suppressed the cholesterol oxidation product-induced neuronal apoptosis by inhibiting NF-κB activation in vitro [47], and attenuated proteasome inhibition-induced neuronal apoptosis by suppressing mitochondria-dependent and BID-dependent pathways of apoptosis in differentiated PC12 cells [48].

The protective effect of TAX against CIS-induced oxidative stress and inflammatory response in the kidney is likely to be attributed, at least partially, to its ability to modulate Nrf2/HO-1 pathway. Nrf2 plays an essential role in the regulation of basal and inducible expression of a plethora of antioxidant and cytoprotective genes [9]. Nrf2 activation has been reported to ameliorate chemotherapeutic agents-induced kidney injury through suppression of excessive ROS generation, oxidative damage, and inflammation [19,21,49,50]. The beneficial role of Nrf2 is supported by the study of Liu et al. [14], which showed that Nrf2 deficiency increased the susceptibility to both ischemic and nephrotoxic AKI in mice. Therefore, Nrf2-activating compounds could be of significant therapeutic benefit against nephrotoxicity induced by CIS. Herein, TAX upregulated Nrf2 and HO-1 significantly in the kidney of CIS-intoxicated mice. These findings added support to previous studies showing the involvement of Nrf2 in mediating the pharmacological effects of TAX. For instance, TAX upregulated Nrf2 and prevented oxidative injury in human RPE [24] and D-galactose-administered mice [51]. Apart from the attenuation of ROS, Nrf2 and HO-1 can directly inhibit NF-κB signaling and the inflammatory response and activate anti-inflammatory mediators, thereby regulating the inflammatory cascade [52].

## 4. Materials and Methods

### 4.1. Animals and Treatments

Thirty male Swiss mice weighing 23–25 g were used to assess the protective effect of TAX against CIS-induced nephrotoxicity. The animals were housed in appropriate cages at standard conditions and a 12-hour light/dark cycle and were provided with food and water *ad libitum*. The animal handling and experimental protocol were approved by the ethical committee of animal experiments at University of Hafr Al-Batin (Ref. No. 0041-1443). The mice were acclimatized for seven days before their allocation into five groups (*n* = 6). Group I served as control and received 0.5% carboxymethyl cellulose (CMC) via oral gavage for 10 days and a single intraperitoneal (i.p) injection of physiological saline at day 7. Group II mice received 50 mg/kg TAX [53] in 0.5% CMC orally for 10 days and an i.p injection of physiological saline at day 7. Mice in groups III, IV, and V received a single i.p. injection of CIS (20 mg/kg) [54] at day 7. Group III mice received 0.5% CMC orally for 10 days and group IV and V animals received 25 mg/kg and 50 mg/kg TAX, respectively, via oral gavage for 10 days. TAX and CIS were supplied by Sigma (USA).

Twenty-four hours after the last treatment, the mice were anesthetized with ketamine/xylazine and blood was collected via cardiac puncture. The blood was left to coagulate and then centrifuged to separate serum. The animals were sacrificed and immediately dissected and both kidneys were excised and washed in cold phosphate buffer (PBS). Samples from the kidneys were immediately immersed in 10% neutral-buffered formalin for histopathological and immunohistochemical examination. Other samples were homogenized (10% *w*/*v*) in cold Tris-HCl buffer (pH = 7.4), centrifuged, and the clear supernatant was kept at −80 °C for biochemical assays.

### 4.2. Assay of BUN, Creatinine, and Pro-Inflammatory Cytokines

Serum BUN and creatinine were assayed using Spinreact (Girona, Spain) kits and the pro-inflammatory cytokines (TNF-α and IL-6) were determined using ELISA kits supplied by R&D Systems (Minneapolis, MN, USA). All assays were conducted as instructed by the manufacturers.

### 4.3. Assay of MDA, NO, and Antioxidants

MDA was determined as previously described by Ohkawa et al. [55] and NO was determined using Griess reagent [56]. Activities of SOD [57], CAT [58] and HO-1 [59], and GSH content [60] were determined in the kidney homogenate of both the control and treated mice.

### 4.4. Histological and Immunohistochemical (IHC) Examination

The formalin-fixed specimens were dehydrated, cleared in xylene, embedded in paraffin, and sectioned into 5-μm slices using a rotary microtome. After deparaffinization and rehydration, the sections were subjected to H&E staining for routine histopathological examination [61]. Histopathological changes were observed using light microscopy and evaluated in a blinded manner by a histopathologist. Tissue damage score was evaluated on 4 points grading score involving congestion and hemorrhage, degeneration, necrosis, and inflammation. For IHC, the deparaffinized and hydrated sections were treated with 0.05 M citrate buffer (pH 6.8) for antigen retrieval followed by 0.3% hydrogen peroxide. The nonspecific antigen-antibody binding was blocked through the addition of normal serum for 20 min. The sections were washed in PBS and probed with anti-NF-κB p65 (ThermoFisher, Waltham, MA, USA; 1:100 dilution), anti-Bax (Abcam, Cambridge, MA, USA; 1:100 dilution), anti-Bcl-2 (Abcam, USA; 1:100 dilution), anti-cleaved caspase-3 (ThermoFisher, USA; 1:100 dilution), and anti-Nrf2 (ThermoFisher, USA; 1:100 dilution) overnight at 4 °C. After washing in PBS, the slides were incubated with the secondary antibodies, and diaminobenzidine was used for color development [62]. The sections were counterstained with Mayer’s hematoxylin, examined under a light microscope. The staining labelling indices of the anti-caspase-3 and anti-NF-κB p65 antibodies were presented as a percentage of positive expression in a total 1000 cells. The immunostaining intensity of anti-Bcl-2 and anti-Nrf2 antibodies was determined through percent of positive area using ImageJ (NIH, Bethesda, MD, USA).

### 4.5. Assessment of the Impact of TAX on CIS Cytotoxicity

HepG2 cells were grown in PRMI-1640 supplemented with 1% penicillin/streptomycin, 10% fetal bovine serum (FBS), and 1% glutamine at 37 °C and 5% CO_2_. The confluent cells were trypsanized and seeded into 96-well plates at a density of 10^4^ cells/well. Different concentrations of CIS and/or TAX were added to the cells which were incubated for 48 h. MTT assay was used for the assessment of cell viability as previously reported [63].

### 4.6. Statistical Analysis

GraphPad Prism 8 software was used for all the statistical analysis. The results are represented as mean ± SEM. Statistical significance among groups was determined using analysis of variance (ANOVA) followed by Tukey’s test. *p* values < 0.05 were considered statistically significant.

## 5. Conclusions

**Figure 9:**
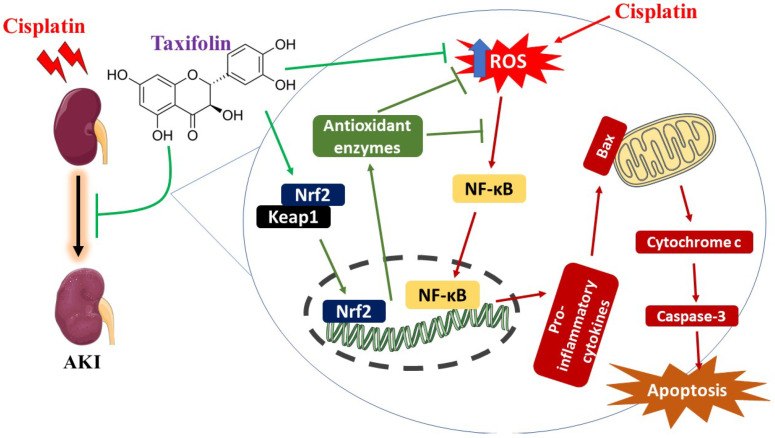
A schematic diagram illustrating the protective effect of taxifolin against cisplatin-induced AKI. Cisplatin induces oxidative stress and activates NF-κB, leading to the release of pro-inflammatory cytokines and apoptosis. Taxifolin mitigated cisplatin-induced acute kidney injury (AKI) by upregulating Nrf2 and preventing oxidative stress, inflammation, and apoptosis.

The findings of this study provide evidence on the protective effect of TAX against the renal complications of CIS. TAX showed a renoprotective effect mediated via modulation of oxidative tissue injury, inflammatory response, and cell death, and upregulation of Nrf2/HO-1 signaling (Figure 9). Therefore, TAX might represent a promising candidate to alleviate kidney injury in patients undergoing CIS chemotherapy, pending further investigations to explore the exact underlying mechanism(s).

## Data Availability

Data is contained within the article.
